# Prediction of the Superficial Heat Source Parameters for TIG Heating Process Using FEM and ANN Modeling

**DOI:** 10.3390/e21100954

**Published:** 2019-09-29

**Authors:** Joanna Wróbel, Adam Kulawik

**Affiliations:** Institute of Computer and Information Sciences, The Faculty of Mechanical Engineering and Computer Science, Czestochowa University of Technology, Dabrowskiego 73, 42-201 Czestochowa, Poland; adam.kulawik@icis.pcz.pl

**Keywords:** artificial neural network, finite element method, TIG welding

## Abstract

The basic problem of the numerical model’s quenching process is establishing the characteristics of the boundary conditions. The existing descriptions of the boundary conditions, which represent the parameters of equipment used in heat treatment processes, do not accurately reflect the actual process conditions. In the present study, the method of choice for superficial heat source parameters for TIG (tungsten inert gas) heating is modeled using artificial neural networks (ANN) and the finite element method (FEM). A comparison of the calculations obtained from the numerical model of non-steady state heat transfer with the results of the experimental studies is presented. The possibility of using ANN to compute the parameters of the boundary conditions for the heating treatment is analyzed. A multilayer feed-forward backpropagation network is developed and trained using value of temperature in the selected nodes obtained from numerical simulation.

## 1. Introduction

The continuous development of knowledge in the range of technical fields has increased demands on modern engineers. Currently, both during the design and implementation of manufacturing processes, special attention is paid to minimizing costs, shortening working time, and improving the efficiency of technological processes. One of the main tools to achieve these goals is optimization. Artificial neural networks (ANN) belong to the dynamically developing field of computational tools called artificial intelligence. They are an attempt to imitate phenomena and processes occurring in nervous systems of living organisms while searching for new technological solutions. Neural networks can be used primarily for the analysis and processing of incomplete disrupted measurement data, their prediction, classification, and control issues [1,2,3]. Therefore, neural networks find many applications. It is now easier to list the areas in which they do not exist than all those in which they are applied to.

The aim of this work is to analyze methods and tools of artificial intelligence to support the process of numerical modeling’s quenching phenomena. The hybrid methods applied in the work include the combination of advanced numerical methods, the theory of thermal phenomena, mechanical phenomena, and phase transformations with artificial intelligence tools such as artificial neural networks. The results obtained in this paper are supposed to answer the question of whether artificial intelligence tools are effective to numerically support heat treatment processes. The control of the numerical model will concern the parameters’ identification of the numerical model’s quenching process. The identification model using the artificial neural networks will determine the parameters of the moving heat source for example, velocity, power, size, and depth of the material’s penetration. The purpose of the paper is to demonstrate that based on the temperature distribution, specified by the user, the parameters of the process can be determined. It is also necessary to determine which temperature values (from which area) should be analyzed.

The literature analysis shows that to identify the parameters of the heat source the ANN are not taken into account. In the field of thermal phenomena, the artificial neural network is applied, among others, to calculate hardness, determine phase transformations, or predict the mechanical properties of steel. Lambiase et al. [4] developed an artificial neural network to predict the achieved hardness in laser hardening. The ANN were trained based on the cooling time and temperature history calculated by a 1D analytical model. Pouraliakbar et al. [5] also applied ANN to determine the heat affected zone (HAZ) hardness of pipeline steels. The mass percent of chemical composition and thermomechanical parameters were the input parameters of the network. Khalaj et al. [6] proposed an artificial neural network to obtain the martensite fraction of microalloyed steel. The input values were experimental data obtained from the literature. The author’s used the Matlab ANN toolbox with the backpropagation algorithm training the network. Powar and Date [7] predicted the mechanical properties and phase transformation of the quenching process using ANN. The input parameters were the alloy composition, as well as the parameters of the heat treatment process and hardness. All computation were carried out using Fluent and Matlab tools. In the authors’ early work [8] the influence of the values of temperature on the parameters of the hybrid heat source (combination of the superficial and volumetric source) were determined by the ANN. All calculations were obtained using copyright software. The results presented in above papers showed that the developed models can be used in a practical application in a manufacturing process.

## 2. Finite Element Simulation

In the paper, the ANN was used to calculate the parameters of the superficial heat source. For this purpose, the heat treatment of a steel element was carried out using tungsten inert gas (TIG) heating. On the basis of the experiment described in the earlier paper of [9], the parameters for the numerical model were selected. The experiment’s parameters:

- Plate sheet with a thickness of 3 mm made of medium carbon steel (C45);
- Thorium tungsten electrode WTh20 with a diameter of 2 mm and a tip grinding angle of 60°;
- Shielding gas—Argone 4.6;
- The distance of the electrode from the workpiece—2 mm;
- Negative direct current source, the arc current I = 60 A, and heating rate V = 0.0033 m/s.

Getting the right amount of input data is associated with long-term and costly experimental research. Therefore, it was decided to replace data from the experiment with data from the numerical simulation.

**Figure 1:**
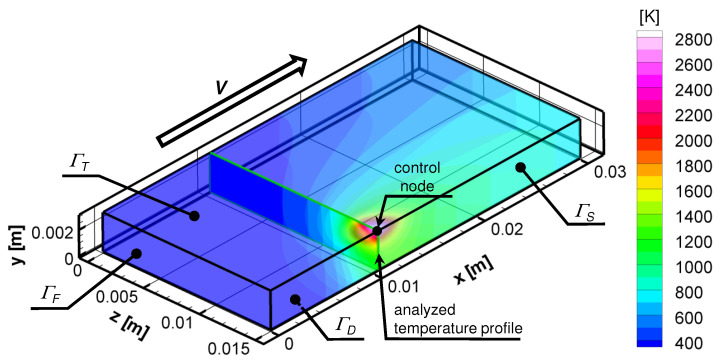
Visualization of example [9].

Three-dimensional simulations were performed to simulate the real process. The computation were carried out for the steel plate with dimensions 0.03×0.003×0.015 m made of C45 steel (Figure 1). The model of thermal phenomena were based on the following equation:

(1) $$\nabla \cdot \left(\lambda \nabla T\right)-\rho c\frac{\partial T}{\partial t}=0$$

In the model, the heating was taken into account using the second-type boundary condition (Neumann): Superficial heat source [10]. The power of the heat source $Q_{N}$ was determined using the equation of effective arc power [11,12]:

(2) $$\begin{array}{c} q_{N}=\frac{Q_{N}}{2\pi R_{N}^{2}}exp\left(-\frac{{\left(x-x_{0}\right)}^{2}+{\left(z-z_{0}\right)}^{2}}{2R_{N}^{2}}\right)\\ Q_{N}=UI\xi ,U=10+0.04I \end{array}$$

The mathematical model was solved by the finite element method in the method of weighted residuals. Eight-point elements with the linear shape function were used.

In the simulation the quenching process for the following conditions were taken into account:

- The initial temperature of the steel element 293 K;
- The Neumann boundary condition in the plane of symmetry ($\Gamma_{S}$) with q = 0 W/m${}^{2}$;
- The Newton–Robin condition on boundaries $\Gamma_{D}$ (lower plane) and $\Gamma_{T}$ (upper plane) with the heat transfer coefficient $\alpha_{\infty }$ for air [13];
- The parameters of the superficial heat source: Heating rate V = 0.0033 m/s (non steady-state), arc current I = 60 A, radius $R_{N}$ = 0.0012 m;
- The Dirichlet condition on $\Gamma_{F}$ (front plane and steady-state);
- The drift velocity (steady-state).

The material properties (λ, ρ, and *c*) were the functions of temperature in the range 273 K–1773 K [14].

**Figure 2:**
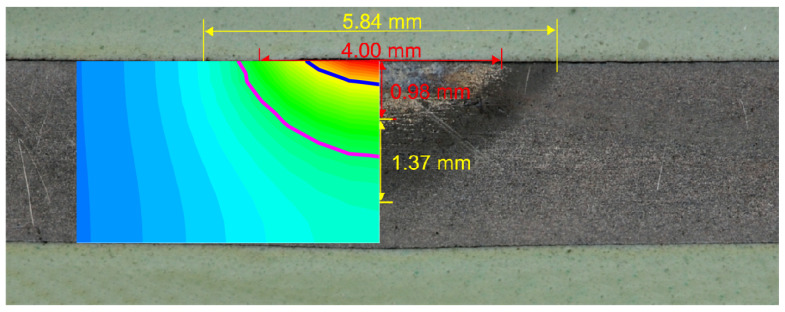
The width and depth of HAZ (heat affected zone) and FZ (fusion zone) obtained from the experiment and numerical simulation (ξ = 0.6).

**Figure 3:**
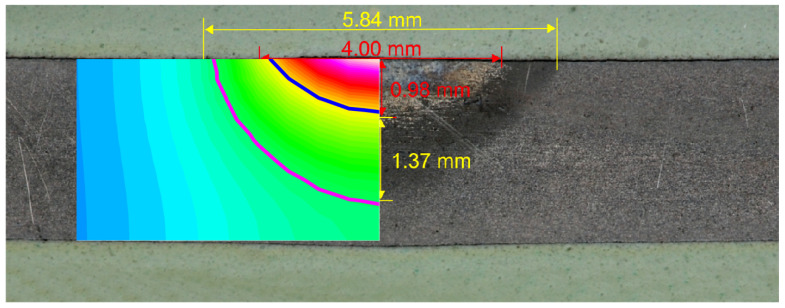
The width and depth of HAZ and FZ obtained from the experiment and numerical simulation (ξ = 0.85).

Several dozen numerical simulations were carried out in order to select the appropriate value of the arc efficiency coefficient. The searched coefficient had to give a comparable with the experiment’s field of heat affected zone (HAZ) and fusion zone (FZ). In the paper the width and depth of HAZ and FZ obtained from numerical calculations and the experiment were compared for two values of the arc efficiency (ξ = 0.6 and ξ = 0.85) (Figure 2 and Figure 3). The first value of arc efficiency was chosen as it is often found in the literature, whereas the second value was selected based on the compatibility with the experiment and fell within the range presented by [15]. The field of HAZ was determined using the $A_{c1}$ temperature (1008 K). The FZ was limited by solidification temperature $T_{s}$ = 1750 K. The size of the heat affected zone and fusion zone was set as the matching parameter.

Good compatibility of the following distributions was obtained: The temperature (fields of HAZ and FZ, see Figure 3), the phase fractions, and the hardness. The obtained results confirmed the correctness of the developed model.

## 3. Artificial Neural Network Modeling

The values of the arc current and velocity of the welding head are only a few parameters, affecting the field of the heat affected zone and fusion zone. Achieving the appropriate size of zones is often associated with many expensive and time-consuming experiments. In this paper, a system to support the selection of the parameters of the quenching process based on ANN was presented. The numerical simulations were performed assuming that the heat source model well reflects the real conditions.

In the presented model, it was assumed that the neural network was trained using data obtained from the solution of the steady-state heat transfer equation. In order to determine the training and testing sets, the 2000 numerical simulations of the heat treatment for a steel element (Figure 1) were carried out. Just as in Section 2, conditions were assumed. The drift velocity in stationary task corresponded to the velocity of the heat source in the non-stationary task.

The quenching was modeled by a superficial heat source with a gaussian distribution, Equation (2). The power and velocity of the heat source were taken based on the values from the experiment. These parameters were changed randomly within the appropriate ranges: V∈[0.0025,0.0058] m/s, and $Q_{N}\in \left[420,860\right]$ W. Whereas, the radius of the heat source was chosen from the range $R_{N}\in \left[0.0002,0.003\right]$ m. The 2000 numerical simulations were carried out, assuming that the maximal temperature on the top surface of the element (control node, see Figure 1) should be in the range of [1000,4000] K. The received data from the simulation were divided into two equal parts and assigned to the training and testing sets. The values of temperature in the z-plane were extracted from the received data (analyzed temperature profile, see Figure 1). The values of temperature were the input data of the ANN. Meanwhile, the power, radius, and velocity of the heat source were the output parameters of the network.

In order to check the possibility of limiting the number of input parameters, the four configurations of ANN were considered (Table 1). The possibility of eliminating parameters that do not have a significant impact on the training process is particularly important when the training data are obtained from time-consuming and expensive experiments.

**Figure 4:**
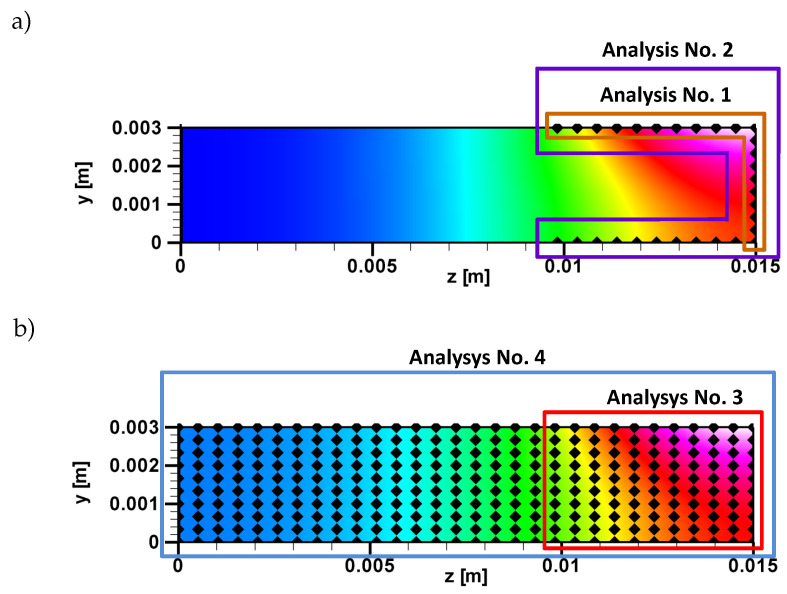
Location of control nodes: (**a**) Analysis No. 1 and No. 2, (**b**) analysis No. 3 and No. 4.

The location of the control nodes for each analysis is shown in Figure 4. A multi-layer perceptron (MLP) network with two hidden layers was used for the calculations. The number of neurons in each layer varied depending on the type of analyzed configuration (Table 1). The training process of MLP network was carried out with the backpropagation algorithm with a momentum term. To training neural network using the backpropagation algorithm, the following steps are required: Preparation of input and output data, generation of initial weights, network architecture, activation function, training of ANN, testing of ANN, and obtaining results [1,2,3]. It was assumed that for all analyzed configurations, the momentum coefficient was equal α = 0.76 and learning coefficient −η = 0.03.

## 4. Results and Discussion

**Figure 5:**
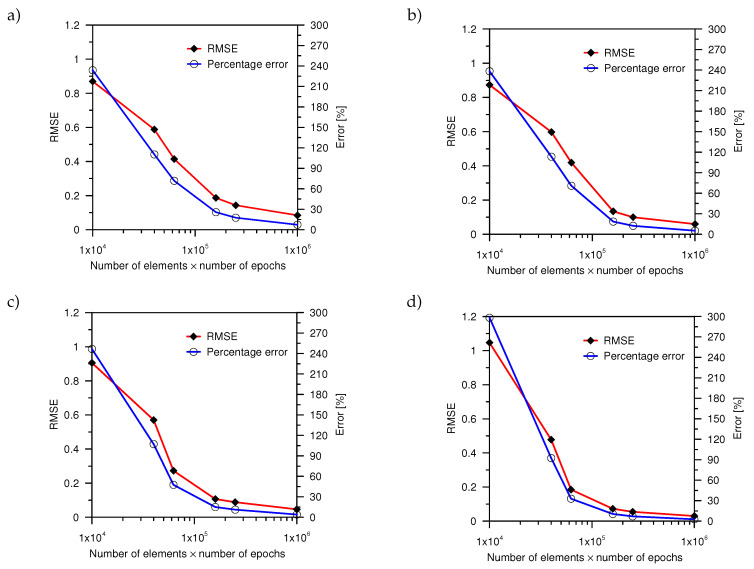
The comparison of the percentage and RMS (root-mean-square) error for the performed computation: (**a**) Analysis No. 1, (**b**) analysis No. 2, (**c**) analysis No. 3, and (**d**) analysis No. 4.

The analysis was carried out for the four cases of the nodes localization, two on the edge in the area of highest changes of temperature, and two in the cross section. The data were divided into two equal sets: Training (1000 samples) and testing (1000 samples). The validation set was not included due to the earlier determination of the ANN structure. The training process was carried out for *n* epochs (n={100,200,240,400,500,1000}). To determine the effectiveness of the ANN, the values of the root-mean-square error (RMSE) and percentage error were used. The values of the errors were obtained on the basis of the 10 series of calculations (Figure 5).

The percentage error, obtained during the training process was comparable for 1 and 2 of the analyzed network configurations. To obtain a value of an error smaller than 10% for the output value, the minimum size of the training set for 1, 2, and 3 analysis must amount to 1,000,000 elements (1000 elements and 1000 epochs). The use of a set for such a number of elements is connected with the necessity of extending training time. On the other hand, for the four configurations of the network, 160,000 elements (400 elements and 400 epochs) was enough to obtain an error below 10%. As mentioned earlier, the important information is the possibility of limiting the number of input data. The use of three network configurations with a training set size of 250,000 (500 elements and 500 epochs) will allow the limit of the input parameters and obtain an error of 11%.

**Figure 6:**
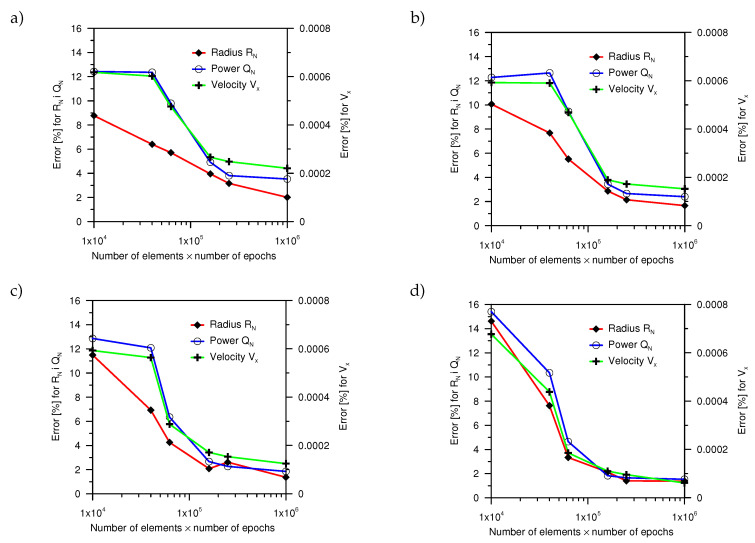
The mean percentage error for the performed computation: (**a**) Analysis No. 1, (**b**) analysis No. 2, (**c**) analysis No. 3, and (**d**) analysis No. 4.

After the training process, the neural network was able to determine the parameters of the boundary conditions for the testing data, which was not presented during the training process. For the determined network weights, for each number of elements in the learning set and the number of epochs, a numerical test was carried out for the data from the testing set. The values of the heat source parameters obtained from the numerical simulation were compared with the values determined by ANN (Figure 6).

It was noticed that, for all of the analyzed cases the error of estimating the drift velocity was below 1%. Whereas, the maximum percentage error of estimation of the power and radius of the heat source did not exceed 1.6% for all network configurations. For 62,500 elements (250 elements and 250 epochs) of the training set, this error was below 10%.

In the next test, on the basis of the output parameters of ANN the numerical simulations were performed. The compatibility of the temperature in the control nodes were compared for both methods (numerical simulation and artificial neural network). The difference for the analysis No. 1 and No. 4 were presented. For all configurations, the same input temperature profile was considered.

**Figure 7:**
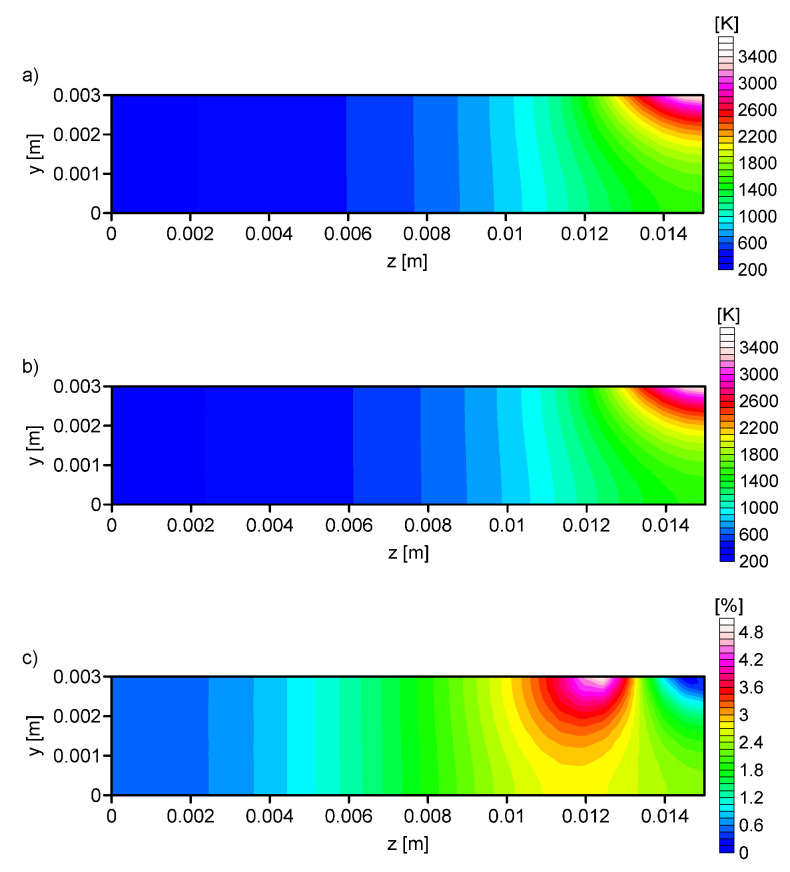
Analysis No. 1: (**a**) The temperature profile obtained from the numerical simulation ($R_{N}$ = 0.00141 m, $Q_{N}$ = 840 W, *V* = 0.002595 m/s), (**b**) the temperature profile calculated on the basis of output data from the ANN ($R_{N}$ = 0.001339 m, $Q_{N}$ = 801.21 W, *V* = 0.002563 m/s), and (**c**) the percentage difference between the required and determined temperature.

**Figure 8:**
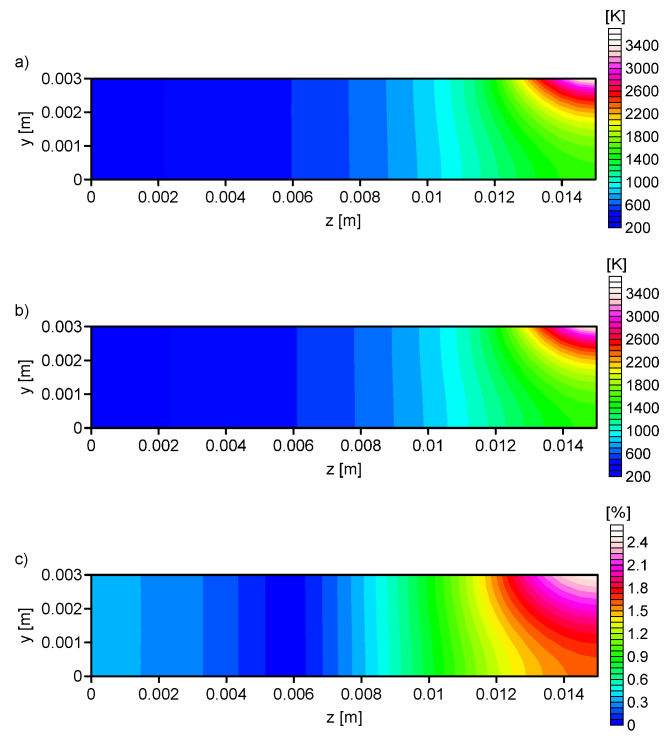
Analysis No. 2: (**a**) The temperature profile obtained from the numerical simulation ($R_{N}$ = 0.001413 m, $Q_{N}$ = 840 W, *V* = 0.002595 m/s), (**b**) the temperature profile calculated on the basis of output data from the ANN ($R_{N}$ = 0.00141 m, $Q_{N}$ = 816.44 W, *V* = 0.002555 m/s), and (**c**) the percentage difference between the required and determined temperature.

**Figure 9:**
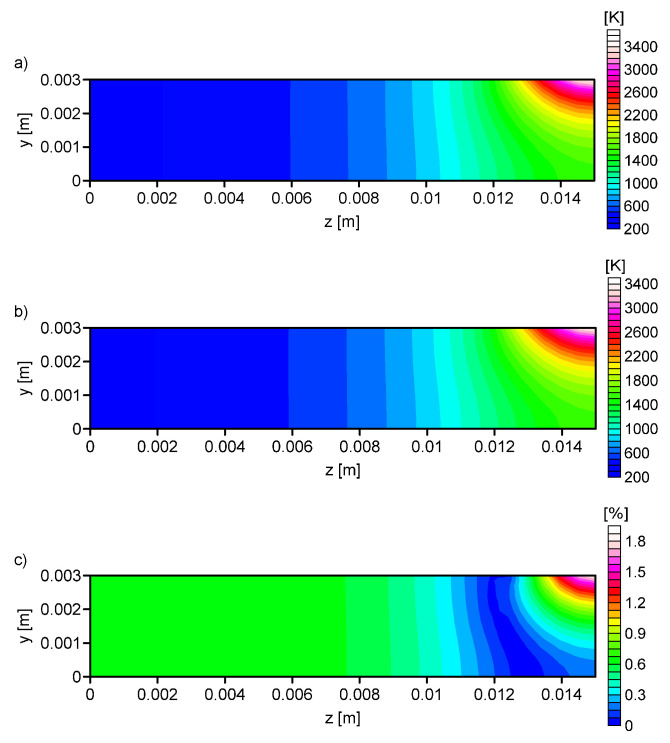
Analysis No. 3: (**a**) The temperature profile obtained from the numerical simulation ($R_{N}$ = 0.00141 m, $Q_{N}$ = 840 W, *V* = 0.002595 m/s), (**b**) the temperature profile calculated on the basis of output data from the ANN ($R_{N}$ = 0.001435 m, $Q_{N}$ = 834.77 W, *V* = 0.002568 m/s), and (**c**) the percentage difference between the required and determined temperature.

**Figure 10:**
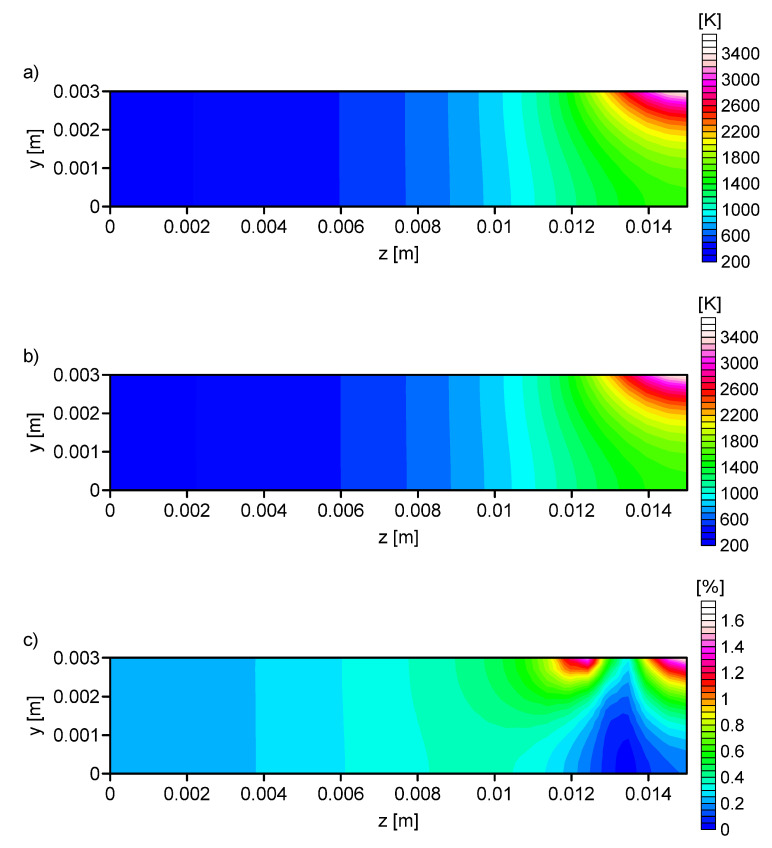
Analysis No. 4: (**a**) The temperature profile obtained from the numerical simulation ($R_{N}$ = 0.00141 m, $Q_{N}$ = 840 W, *V* = 0.002595 m/s), (**b**) the temperature profile calculated on the basis of output data from the ANN ($R_{N}$ = 0.001373 m, $Q_{N}$ = 830.91 W, *V* = 0.0026 m/s), and (**c**) the percentage difference between the required and determined temperature.

Figure 7, Figure 8, Figure 9 and Figure 10 shows the: Temperature profile loaded into the network input (Figure 7a), temperature profile calculated on the basis of output data from the ANN (Figure 7b, Figure 8b, Figure 9b and Figure 10b), and the percentage difference between profiles (Figure 7c, Figure 8c, Figure 9c and Figure 10c). The local percentage error value for the temperature profile in the cross-section was not higher than 5% (Figure 7c, Figure 8c, Figure 9c and Figure 10c).

A comparison of the numerical simulations and experiment results shows that an important problem in the modeling of the thermal phenomena is an appropriate choice of the heat source model and its parameters. The use of a superficial heat source, according to the values of arc efficiency presented in literature [12], caused poor matching between the HAZ and FZ (Figure 3). The applied artificial neural networks allowed the determination of the parameters of the heat treatment process for a given temperature profile. This method allows for the easy control of the quenching process, and modification of the heat source parameters for the required field of the HAZ and FZ. The knowledge contained in ANN, obtained from the simulation avoids expensive research. The performed analysis confirmed the possibility of limiting the number of input parameters in the artificial neural network by up to 1/3.

## 5. Conclusions

Based on the obtained results and the accuracy of the artificial neural network, it can be concluded that the trained neural network can select parameters of simulation for modeling the TIG process of medium carbon steels. This approach allowed the control of both the heat affected zone and the fusion zone. The obtained data could also be used to determine the actual process parameters.

Using the artificial neural network allows, after proper training, for one to determine the parameters of the heat treatment process. In the presented study, the application of the ANN allowed the determination of the parameters of the superficial heat source for a limited number of network input. A total of four network configurations were considered. It was noted that the percentage error decreased when the number of training inputs increased. For the fourth network configuration this error was ≈1.6% and the observed maximum error value applied to a small area (Figure 10d). The accuracy of the results achieved from neural networks was satisfactory for 110 neurons in the input layer. On the basis of the obtained values of error for four network configurations, it can be concluded that for the training of ANN, the data was needed not only from the surface of the quenching element but also from the interior of the element. Therefore, data in the training sets must come from accurate numerical simulations.

All computation results presented in the paper were performed using the authors’ own software. The implementation was done by the authors of this paper in C++ language on the Visual Studio 2010 platform. The functions of linear algebra were taken from the Intel^®^ Math Kernel Library 11.0.

## Figures and Tables

**Table 1:** Analyzed configurations of the artificial neural network.

ANN Structure- Analysis No.	Number of Neurons in Layer			
	Input ($N_{0}$)	I Hidden ($N_{1}$)	II Hidden ($N_{2}$)	Output
1	20	12	6	3
2	30	20	10	3
3	110	50	20	3
4	300	150	30	3

## Abbreviations

*T*	Temperature [K]
λ	Thermal conductivity coefficient [W/(mK)]
ρ	Density $\left[kg/m^{3}\right]$
*c*	Thermal capacity [J/(kgK)]
*t*	Time [s]
$q_{N}$	Heat flux on boundary (II-type boundary condition) $\left[W/m^{2}K\right]$
$Q_{N}$	Power of the superficial heat source [W]
$R_{N}$	Radius of the superficial heat source [m]
$x_{0},z_{0}$	Coordinates of the centre of heat source
*U*	Arc voltage [V]
*I*	Arc current [A]
ξ	Arc efficiency
